# Role of Global and Local Topology in the Regulation of Gene Expression in *Streptococcus pneumoniae*

**DOI:** 10.1371/journal.pone.0101574

**Published:** 2014-07-14

**Authors:** María-José Ferrándiz, Cristina Arnanz, Antonio J. Martín-Galiano, Carlos Rodríguez-Martín, Adela G. de la Campa

**Affiliations:** 1 Unidad de Genética Bacteriana, Centro Nacional de Microbiología, Instituto de Salud Carlos III and CIBER Enfermedades Respiratorias, Madrid, Spain; 2 Consejo Superior de Investigaciones Científicas, Madrid, Spain; Centers for Disease Control & Prevention, United States of America

## Abstract

The most basic level of transcription regulation in *Streptococcus pneumoniae* is the organization of its chromosome in topological domains. In response to drugs that caused DNA-relaxation, a global transcriptional response was observed. Several chromosomal domains were identified based on the transcriptional response of their genes: up-regulated (U), down-regulated (D), non-regulated (N), and flanking (F). We show that these distinct domains have different expression and conservation characteristics. Microarray fluorescence units under non-relaxation conditions were used as a measure of gene transcriptional level. Fluorescence units were significantly lower in F genes than in the other domains with a similar AT content. The transcriptional level of the domains categorized them was D>U>F. In addition, a comparison of 12 *S. pneumoniae* genome sequences showed a conservation of gene composition within U and D domains, and an extensive gene interchange in F domains. We tested the organization of chromosomal domains by measuring the relaxation-mediated transcription of eight insertions of a heterologous P*_tc_cat* cassette, two in each type of domain, showing that transcription depended on their chromosomal location. Moreover, transcription from the four promoters directing the five genes involved in supercoiling homeostasis, located either in U (*gyrB*), D (*topA*), or N (*gyrA* and *parEC*) domains was analyzed both in their chromosomal locations and in a replicating plasmid. Although expression from the chromosomal P*_gyrB_* and P*_topA_* showed the expected domain regulation, their expression was down-regulated in the plasmid, which behaved as a D domain. However, both P*_parE_* and P*_gyrA_* carried their own regulatory signals, their topology-dependent expression being equivalent in the plasmid or in the chromosome. In P*_gyrA_* a DNA bend acted as a DNA supercoiling sensor. These results revealed that DNA topology functions as a general transcriptional regulator, superimposed upon other more specific regulatory mechanisms.

## Introduction

In all organisms, DNA is dynamically compacted in a way optimal for DNA replication, chromosome segregation and gene expression. The shape and spatial extension of the chromosomal DNA of bacteria, the nucleoid, is determined both by DNA supercoiling and by the nucleoid-associated proteins. Many of these proteins also alter DNA topology by bending, wrapping or bridging, and in addition, they influence transcription by constraining supercoils. Although several nucleoid-associated proteins have been characterized in the Gram-negative bacteria *Escherichia coli*, very few have been described in Gram-positives [1]. In this scenario, it is assumed that transcription in bacteria is controlled by a combination of chromosomal topology, promoter DNA sequences, and *trans*-acting protein regulators. These regulators could either facilitate or inhibit the interaction of the RNA polymerase with specific promoter regions [2], or target several genes, such as those of the nucleoid-associated proteins [1].

In bacteria, DNA supercoiling level is mainly maintained by the homeostatic activities of DNA topoisomerases that relax DNA, and by the DNA gyrase, which introduces negative supercoils. DNA topoisomerases catalyze the inter-conversions of different topological forms of DNA and thus solve the topological problems associated with replication, transcription, and recombination [3]. Based on their DNA cleavage pattern, they are divided into two classes. Those that cleave only one DNA strand are classified as type I, whereas those that cleave both strands, generating a double-strand break, are classified as type II. In *E. coli*, transcription of the topoisomerase I gene (*topA*) increases when supercoiling rises [4], in opposition to that of DNA gyrase genes (*gyrA*, and *gyrB*), which increase after DNA relaxation [5]–[7]. Likewise, changes of *gyrA* and *gyrB* expression in response to relaxation have been also observed in other bacteria [8], [9]. Besides the regulation by supercoiling level, it is not fully understood how transcription of DNA topoisomerases is controlled. In *E. coli*, expression of gyrase and topoisomerase I genes is subjected to homeostatic regulation by nucleoid-associated proteins [10], which control DNA supercoiling [11]. Nevertheless, these regulatory mechanisms may not apply to *Streptococcus pneumoniae*, which lacks most of the nucleoid-associated proteins found in *E. coli*. In addition, in several bacterial species, the DNA supercoiling level is affected by a variety of environmental conditions. In addition chromosome replication as well as stages of growth and infection affect DNA supercoiling [12]–[14]. Recent transcriptomic studies have shown that changes in DNA supercoiling also have an effect on global genome transcription, as observed in *E. coli* [15], [16], *Haemophilus influenzae* [17] and *S. pneumoniae* [18]. In *S. pneumoniae*, treatment with the GyrB inhibitor novobiocin [19], [20] causes chromosome relaxation and a transcriptional response affecting all their DNA topoisomerases: up-regulation of gyrase and down-regulation of topoisomerases I and IV [18]. DNA relaxation also triggers a global transcriptional response affecting about 14% of the genome. A majority (>68%) of responsive genes are closely positioned forming 15 gene clusters (localized either in up- and down-regulated topological domains), which show a coordinated response that surpass the operon organization [18]. In this way, the chromosome of *S. pneumoniae* is organized into four kinds of domains: up-regulated (U), down-regulated (D), non-regulated (N), and flanking (F) domains. F domains are located adjacent to D-domains, have a remarkably high AT content >65%, and could play a structural role in the maintenance of DNA topology.

The aim of the present study was to determine a precise map on the organization of the *S. pneumoniae* chromosome in topological domains, and on the mechanism by which this organization functions as a main global factor controlling gene expression. To this end, general transcriptional and conservation features of the domain organization were evaluated among 12 pneumococcal strains. In addition, several genetic constructions were made to analyze gene expression from cognate (pneumococcal) or heterologous promoters, in single (chromosome) or multiple (plasmid) gene dosages. Expression of a foreign P*_tc_cat* cassette in eight different chromosomal locations was analyzed by means of quantitative real-time PCR (qRT-PCR). The activity of the promoters of the pneumococcal DNA topoisomerase genes, in their chromosomal locations and in replicating plasmids, together with the role of a DNA curvature at the promoter of the topology-controlling gene *gyrA*, was studied.

## Results

### Distinct domains show different conservation of gene composition and expression tendencies

**Figure 1:**
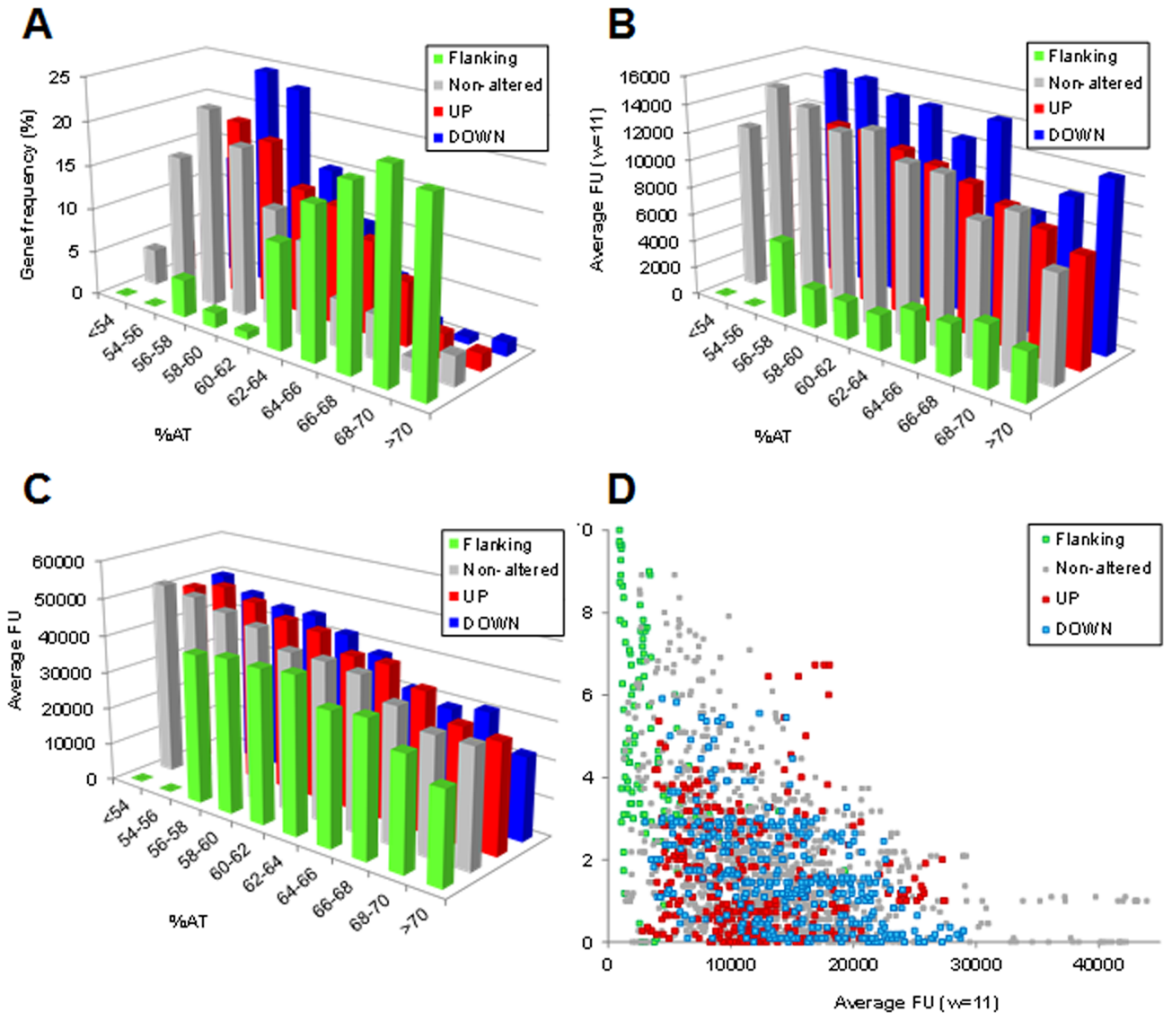
Domains show conservation and expression tendencies. (A) Relationship between gene frequency and AT content in microarrays hybridized with cDNAs obtained from total RNA. (B) Relationship between average FU and AT content in microarrays hybridized with cDNAs obtained from total RNA. A window of 11 genes (average value on the middle one) was applied for FU values. Only coding regions were considered in the AT calculations. (C) Relationship between FU value and AT content in microarrays hybridized with cDNA obtained from total DNA, the median of about 32 microarray spot values was calculated and the mean of medians from two replicates considered. (D) Relationship between the gene lack index and FU. Genes were considered equivalent to R6 counterparts when their polypeptide products shared ≥80% identity over ≥80% of the sequence length in other *S. pneumoniae* strains with complete genome sequence: 70585, ATCC 700669, CGSP14, D39, G54, Hungary19A-6, JJA, P1031, Taiwan19F-14, TCH8431/19A and TIGR4. An 11-gene window (roughly equivalent to 10 Kb) was applied. Misleading continuum of sharp peaks or poorly defined peaks were obtained when narrower or wider windows were applied, respectively.

We explored the global nature of the domains at transcriptional and evolutionary levels. Microarray fluorescence units (FU) raw values at time 0 min (OD_620nm_ = 0.4), without novobiocin treatment, were studied as an indirect measure of gene transcription levels under basal conditions. In order to inspect gene contexts, AT content (%AT) and FU were compared. A significant and inverse relationship (*P*<10^−178^; R^2^ = 0.327) was found between %AT and FU when genes from all domains were considered (U:363, D: 393, F:86, and N:1195 genes). Under basal conditions, genes of D and U domains showed FU values 13% higher and 8% lower, respectively, than the genome average (12581 FU). On the other hand, genes of the F domains, which showed the highest AT content (Figure 1A), also showed the lowest FU levels, being >3-fold lower than the genome average, with ∼85% genes having FU below 6000 units (Figure 1 B). This inverse relation between AT content and FU could be a technical artifact derived from the higher stability of the hybridization of high GC sequences in the arrays. When compared with non-altered genes in the same %AT range, flanking genes were significantly under-expressed (p-values: 3×10^−4^, 3×10^−3^ and 8×10^−5^, two-tailed unpaired t-test, in the %AT ranges of 60–62, 62–64, and 64–66, respectively). This demonstrates that domain type is more important than AT content in determining the FU value. In addition, to double check the basal dependence of AT content for fluorescence, chromosomal DNA was utilized in a parallel experiment as template to generate cDNA and hybridize to microarrays. Within ranges of AT content, FU values for the four classes is much more comparable when the template was DNA than when it was RNA (Figure 1 C). This confirms that the lower FU values obtained for genes of F domains is due to lower transcription and not to less efficient hybridization mediated by a minor AT content.

A comparison of the 12 *S. pneumoniae* genome sequences available provided evidence for the conservation of the U and D domains. A gene-lack index at the species level, defined as the number of genomes in which a gene is present divided by the total number of strains analyzed, was derived. The relationship between the gene-lack index and transcription efficiency is shown in Figure 1D. The gene-lack index was lower on average in U (1.51) and D (1.65) domains than in the genome (1.91). In sharp contrast, F domains showed very high gene-lack indexes (average 4.66), indicating an extensive gene interchange in these domains. Mainly genes with a low expression (low FU value) have a high gene-lack. Despite this high rate, more than half of F-domains genes still remained in all strains.

### The P*_tc_cat* gene cassette shows a location-dependent transcription in response to DNA relaxation

**Figure 2:**
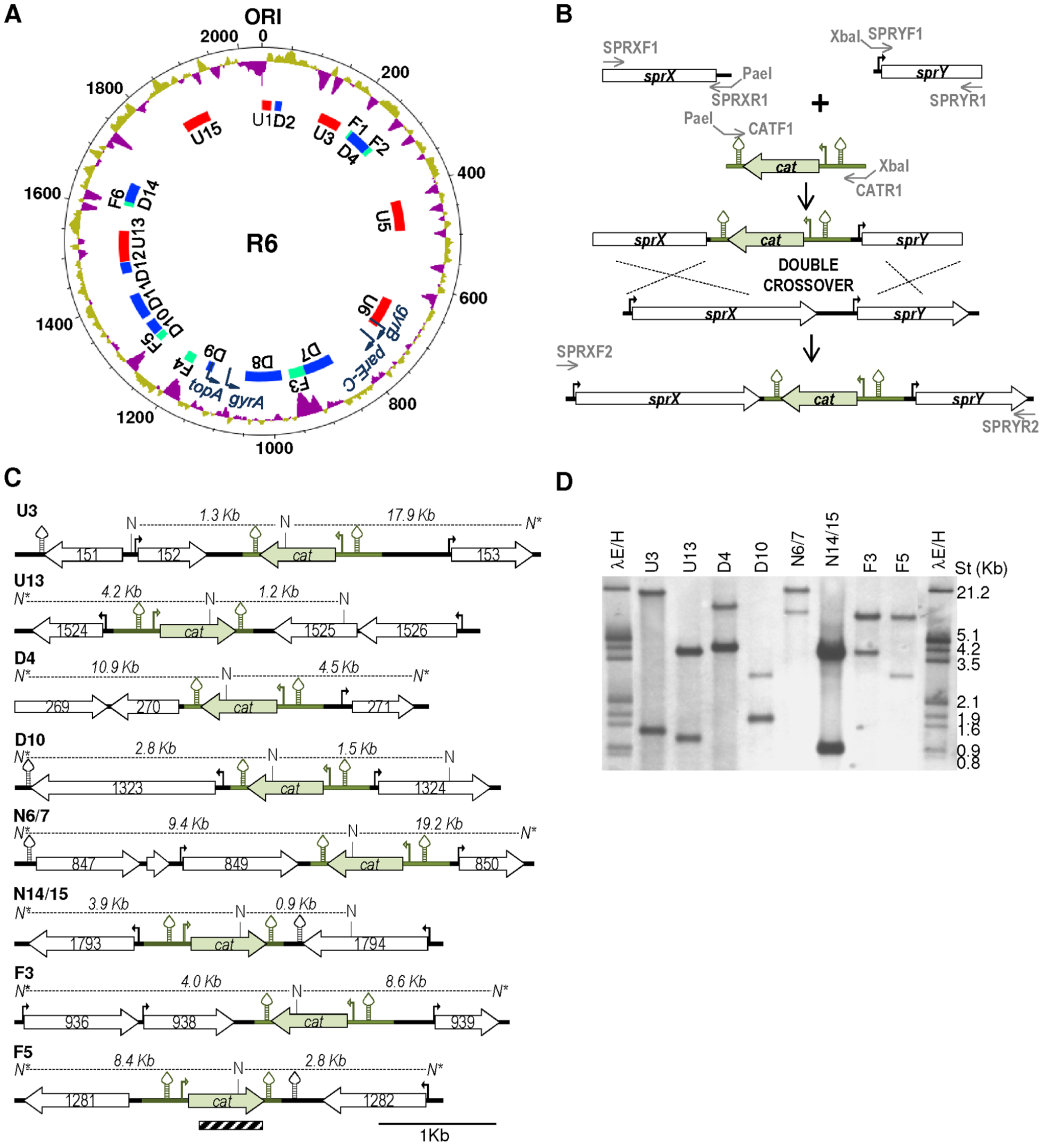
Construction of R6-derivative strains with P*_t_* _**c**_ ***cat*** **insertions.** (A) Organization of the *S. pneumoniae* R6 chromosome in topology-related domains. Circles (outside to inside) represent: % GC (values above the average in purple); DNA topoisomerase genes (dark blue curved arrows); topology-responsive domains. The chromosome is organized in domains up-regulated (U, red boxes) or down-regulated (D, blue boxes) in response to DNA relaxation; and flanking regions (F, green boxes). Representation has been performed with Artemis and DNA plotter software at www.sanger.ac.uk/resources/software/artemis [45]. (B) Insertion of the P*_t_*_c_ *cat* cassette in diverse supercoiling domains. The *cat* cassette (green drawing), promoters (curved arrows) and transcription terminators (stem and loop structures) and oligonucleotides with their restriction targets are indicated. (C) Gene organization of the strains indicated. R6 genes (spr numbering) are indicated by white arrows. The *cat* cassette, promoters and transcription terminators are shown as in B, N, NcoI targets and predicted sizes of fragments, N* indicate that the target is outside the region shown. Distances between NcoI targets are shown above the dashed lines. Stripped box at the bottom of the figure represents the *cat* probe used for Southern analysis and its position inside the *cat* gene. (D) Southern blot hybridization of R6-CAT strains. Chromosomal DNA from the strains was cut with NcoI, separated by agarose gel electrophoresis, transferred to a nylon membrane and hybridized with the biothynilated *cat* probe shown in C.

To check topology-related transcription, the heterologous P*_tc_cat* gene cassette, which derives from the *Staphylococcus aureus* pC194 plasmid [21], [22], and codes for chloramphenicol-acetyl-transferase, was inserted into different topological domains (Figure 2 A). This P*_tc_cat* cassette carried the *cat* gene under the control of two promoters (P*_t_* and P*_c_*) and is flanked by two transcriptional terminators, its nucleotide sequence and relevant features are shown in Figure S1. Insertions of P*_tc_cat* were made into intergenic regions of at least 190 bp. A two-step scheme was followed for all constructions as described in materials and methods (Figure 2 B). In the first step, two fragments of contiguous genes flanking the selected site of insertion were ligated to P*_tc_cat*. In the second step, the *sprX*−P*_c_cat*−*sprY* linear fragments were introduced by homologous recombination into the chloramphenicol-susceptible R6 strain. Eight different strains, carrying individual different insertions, two in each type of domain, were produced (Figure 2 C): U3 and U13; D4 and D10; F3 and F5; N6/7 and N14/15. To prevent transcription read-through from adjacent regions, in addition of the two transcriptional terminators present in P*_tc_cat*, the transcriptional direction of each cassette was opposite, by design, to that of their upstream adjacent genes. Hybridization with a *cat* probe (Figure 2 D) confirmed both chromosomal location, and a single insertion of P*_tc_cat* per strain. The sizes of the labeled fragments (Figure 2 D) corresponded with the expected ones for each strain (Figure 2 C). In addition, the insertions were confirmed by sequencing as described in materials and methods. All the strains shared the same chloramphenicol MIC of 5 µg/ml, suggesting that under basal conditions the expression of *cat* is occurring indeed from their own promoters and independent of chromosomal location.

**Figure 3:**
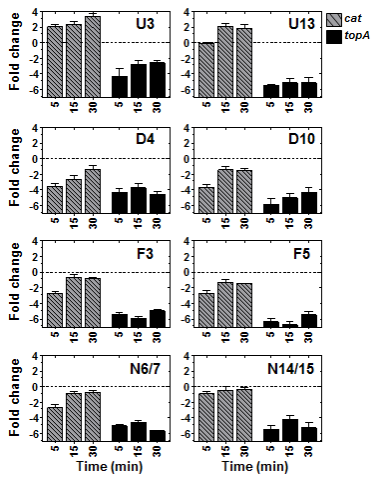
Topology-dependent transcription of P*_tc_cat* is dependent on its chromosomal location. Transcriptional response to DNA relaxation by novobiocin measured by qRT−PCR. Exponentially growing cultures of the R6-CAT strains at OD_620nm_ = 0.4 were treated with novobiocin at 10× MIC. Total RNA was isolated; cDNA was synthesized and subjected to qRT−PCR. To normalize the three independent replicate samples, values were divided by those obtained of an internal fragment of the 16SrDNA gene. These normalized values were made relative to those obtained at time 0 min. Fold change represented the log2 mean of qRT-PCR values of three independent replicates ± SEM.

The eight R6-CAT strains constructed were treated with the GyrB inhibitor novobiocin [19], [20] at 10× MIC (10 µg/ml). We have previously measured the supercoiling density (σ) values of *S. pneumoniae* cultures treated in the same conditions by analyzing the topoisomer distributions of an internal plasmid. We observed plasmid relaxation, σ values decreased from −0.059 (0 min), to −0.036 (5 min), −0.033 (15 min), and −0.024 (30 min). Relaxation was associated to a homeostatic transcriptional response, which included *gyrA* and *gyrB* up-regulation and *parE*-*parC* and *topA* downregulation [18]. In the present study, the level of transcription of *topA*, which is located in a D domain, was used to assess DNA relaxation. To normalize the qRT-PCR values, they were made relative to those of the 16SrDNA gene, whose transcription level in the diverse strains was essentially constant under novobiocin treatment (Figure S2). The addition of novobiocin caused, as expected [18] down-regulation of *topA* in all strains, providing confirmation of DNA relaxation. Gene expression variations after 5, 15, and 30 min of treatment were measured by qRT-PCR, and values were made relative to the untreated culture (time 0 min). The transcription of P*_tc_cat* was dependent on its chromosomal location, being up-regulated when located in U3 and U13 domains, down-regulated when located in D4 and D10, and almost no regulated when located in the N14/15 region. With respect to the N6/7 and F regions, some down-regulation was observed, especially at 5 min time (Figure 3). All these data support the organization of the chromosome in topological domains, which are reactive to interferences in the chromosomal supercoiling status. It was not necessary to correct for altered gene dosage of *cat* insertions closer to the origin since novobiocin does not lead to higher ori/ter ratio's in *S. pneumoniae* [23].

### Expression from *P_topA_* and *P_gyrB_* differ when located in their natural chromosomal location or in a replicating plasmid

**Figure 4:**
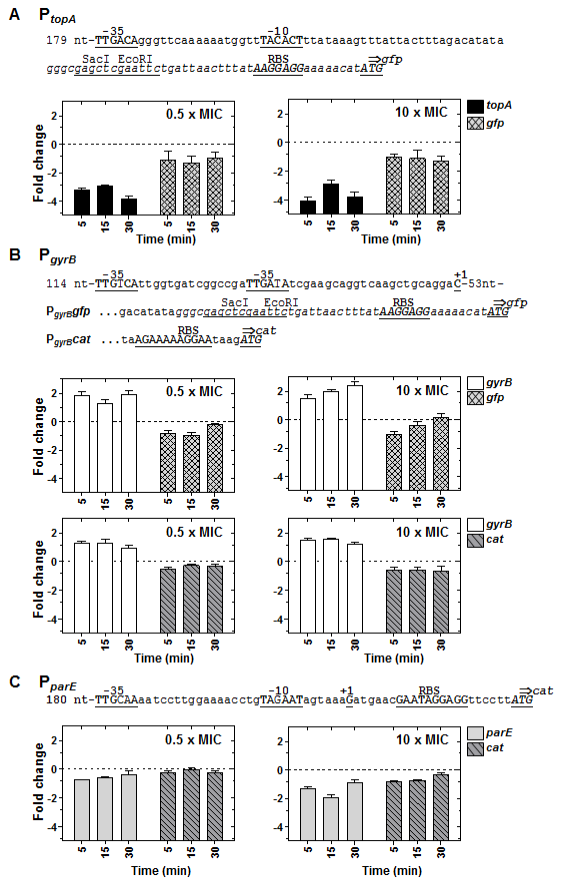
Relaxation-dependent transcription of P*_topA_* and P*_gyrB_* is different in their chromosomal location and in a replicating plasmid and similar for P*_parE_*. (A) A culture of R6 carrying a plasmid with a P*_topA_gfp* fusion was grown until OD_620nm_ = 0.4 and treated with novobiocin at 0.5× MIC and 10× MIC. Samples were processed as described in Figure 3. Results obtained from qRT-PCR analysis at the two novobiocin concentrations indicated are shown. (B) Results obtained with two cultures of R6 one carrying a plasmid with a P*_gyrB_gfp* fusion and the other a plasmid with a P*_gyrB_cat* grown and treated as in A. (C) Results obtained with a culture of R6 carrying a plasmid with a P*_parE_cat* fusion grown and treated as in A. The expression from the promoters in their chromosomal locations (*topA*, *gyrB, parE*) was also determined in the same cultures. Relative values (log2 mean of three independent replicates ± SEM) are represented. To normalize the three independent replicate samples, values were divided by those obtained from internal fragments of the 16S rDNA. These normalized values were made relative to those obtained at time 0 min. The nucleotide sequences of the promoter regions present in the plasmids carrying the fusions are indicated in each case. The −35 and −10 boxes, the +1 mRNA, the ribosome-binding site (RBS), and the ATG initiation codon are indicated in upper case and underlined. Letters in cursive are those present in the pAST vector used for cloning.

Plasmids containing the promoter regions of *topA* (nt −243 to −18, taking the first gene nucleotide as nt 1) or *gyrB* (nt −235 to −16) fused to the *gfp* gene were constructed as described in material and methods and introduced in *S. pneumoniae* R6. P*_topA_* has typical −35 and −10 sequences (Figure 4 A), although its +1 position has not been determined. Unlike other pneumococcal genes, P*_gyrB_* has two canonical −35 boxes and lacks a typical −10 box (Figure 4B). The +1 position has been determined at 70 bp upstream the ATG codon (Figure S3). Transcription from P*_topA_* and P*_gyrB_* was measured by qRT-PCR after DNA relaxation induced by two novobiocin concentrations (0.5× MIC, and 10× MIC). As expected, DNA relaxation caused down-regulation of *topA* and up-regulation of *gyrB* when located in their native chromosomal sites (D9 for *topA* and U6 for *gyrB* in Figure 2A) in both strains (Figure 4A and B). Transcription from P*_topA_* and P*_gyrB_* was also measured in the plasmid *gfp* fusions. P*_topA_* also showed down-regulation in the plasmid (Figure 4 A), although at a lower level (2.4− versus 16.6-fold decrease at 30 min 10× MIC) than in the chromosome. P*_gyrB_* showed up-regulation (about 3−fold) in the chromosome but down-regulation in the plasmid (Figure 4B). The down-regulation of P*_topA_* and P*_gyrB_* was similar in both plasmid constructions. Similar results were obtained in a fusion between P*_gyrB_* and the *cat* gene (Figure 4 B). These results indicate that both *topA* and *gyrB* genes are under a supercoil-mediated complex self-regulation and that the plasmid behaves as a D domain.

### Expression from *P_parE_* is equivalent in their natural chromosomal location and in a replicating plasmid

A plasmid containing the promoter region of the *parE-parC* operon, including positions -215 to the ATG of *parE* fused to the *cat* gene was constructed as described in material and methods and introduced in *S. pneumoniae* R6. P*_parE_* has typical −35 and −10 sequences (Figure 4 C), and its +1 position has been previously determined [24]. Transcription from P*topA*, P*gyrB* and P*parE* was measured by qRT-PCR after DNA relaxation with two novobiocin concentrations (0.5× MIC, and 10× MIC). DNA relaxation caused, as expected, down-regulation of *topA* and *parE*, and up-regulation of *gyrB* when located in their natural chromosomal sites (D9 for *topA*, N6/7 for *parE*, and U6 for *gyrB* in Figure 2 A). The regulation of the P*_parE_cat* fusion in the plasmid was equivalent to that in the chromosome (Figure 4 C).

### Characterization of the sequences involved in the relaxation-dependent transcription of P*_gyrA_*

**Figure 5:**
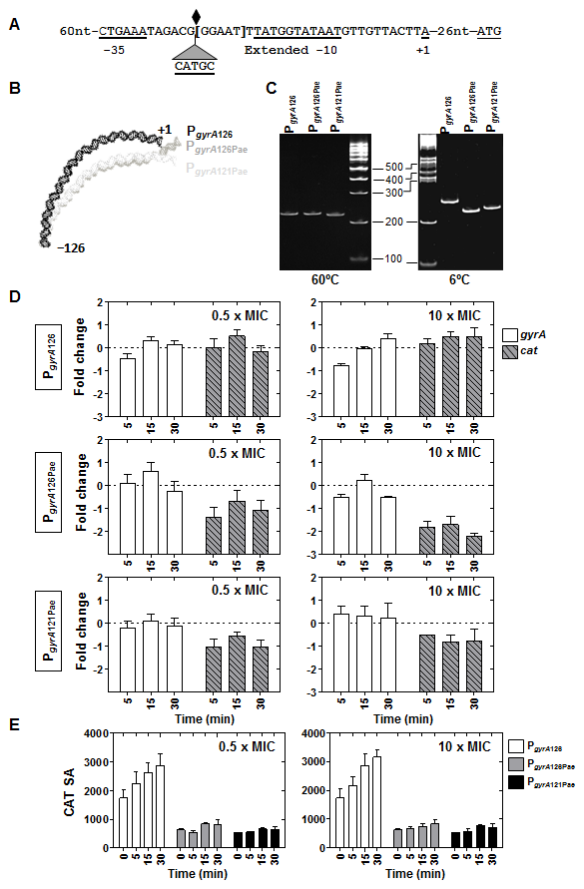
The relaxation up-regulation of P*_gyrA_* depends on a bending. (A) Sequences of wild-type P*_gyrA_*, P*_gyrA_*_126Pae_ and P*_gyrA_*_121Pae_ derivatives. The −35 and extended −10 boxes, the nucleotide in which transcription is initiated (+1), the center of the intrinsic DNA curvature (diamond), and the location of the inserted GATC sequence that creates a PaeI restriction site are indicated. The 5 nucleotides deleted in P*_gyrA_*_121Pae_ are into brackets. (B) Curvature prediction in P*_gyrA_* and P*_gyrA_*_Pae_ by using the model.it program at http//www.icgeb.trieste.it/dna program [46]. (C) Mobility of 232-bp (P*_gyrA_*), 237-bp (P*_gyrA_*_126Pae_) and 232-bp (P*_gyrA_*_121Pae_) fragments in acrylamide gels at 60°C and 6°C to detect DNA curvature. (D) Transcription from P*_gyrA_*, P*_gyrA_*_126Pae_, and P*_gyrA_*_121Pae_. Results obtained from qRT-PCR analysis at 0.5× MIC and 10× MIC novobiocin concentrations are indicated. Cultures were grown and samples processed as described in Figure 3. Relative values (mean of three independent replicates ± SEM) are represented and made relative to those obtained at time 0. Normalization of values was made dividing by those obtained from internal fragments of 16SrDNA gene. € CAT activity measurements detected as described in material and methods. Values represented are the mean of three independent replicates ± SEM. Specific activity, SA, is expressed as nmol acetylated chloramphenicol/mg of protein.

Although *gyrA* is located in an N domain, its transcription is up-regulated by DNA relaxation [18]. We have previously shown that the *gyrA* upstream region carries an intrinsic DNA curvature [25], reducing its mobility in polyacrylamide gels, which can be attributed to the curvature of the helix backbone. Curved DNA molecules have larger cross-sectional areas than normal molecules and require larger pores to migrate through the gel matrix. Therefore, electrophoretically they behave as though they were larger than their true sizes [26]. These anomalously slow electrophoretic mobilities are also due in part to their anomalously slow mobilities in free solution [27]. The low mobility of curved DNA fragments is more evident at low temperatures. In this way, a value for the R^6/60^ factor, the relation between the apparent sizes of the inserts of the plasmids at 6 and 60°C [28] higher than 1, is indicative of curvature. The minimal size of the promoter region maintaining both activity and bending was determined by 5′−nested deletions of the −310 to +1 *gyrA* upstream region fused to *cat* in the pUC18 *E. coli* plasmid. To check the curvature of P*_gyrA_*, the R^6/60^ factor was calculated (Figure S4). The shorter *gyrA* upstream sequence that conserved the curvature (P*_gyrA_*, nt −126 to +1, Figure 5 A) was fused to *cat* into pLS1 plasmid yielding pLGYAC126 (see material and methods). Two constructions were made to eliminate the curvature of P*_gyrA_* in this plasmid. The first consisted in the insertion of a 5−nt CATGC sequence, which created a PaeI target (GCATGC), in the centre of the curvature, yielding plasmid pLGYAC126Pae (Figure 5 A). The second was the deletion of 5-bp (GGAAT) located at the 3′-end of the PaeI target; yielding plasmid pLGYAC121-Pae (Figure 5 A). It was predicted in both constructions the elimination of the intrinsic curvature (Figure 5 B). To test this, fragments containing P*_gyrA_* promoters from pLGYAC126, pLGYAC126Pae, and pLGYAC121-Pae were amplified with specific oligonucleotides, yielding sizes of 232, 237, and 232 bp, respectively. Although these fragments showed equivalent sizes when run at 60°C, their mobility was clearly different when run at 6°C. The 232−bp fragment carrying the wild-type promoter showed a R^6/60^ factor of 1.2, being of 1 for the two other constructions (Figure 5 C). A direct correlation was observed between CAT activity and curvature under basal conditions. The specific activity at time 0 for pLGYAC126 (average ± SD: 1741±525) was approximately 3-fold higher than that for pLGYAC126Pae (average ± SD: 622±83) and pLGYAC121-Pae (average ± SD: 522±17). Then, in P*_gyrA_*, the curvature behaved as an activator *per se*.

To know the role of the curvature in the up-regulation of P*_gyrA_* in response to DNA relaxation, transcription from the chromosomal P*_gyrA_* (Figure 5 D) and from the P*_gyrA_cat* fusions in plasmids were determined by qRT-PCR. While in pLGYAC126 the up-regulation of *cat* was similar to that of the chromosomal *gyrA*, down-regulation of *cat* was observed in plasmids pLGYAC126Pae and pLGYAC121-Pae. The down-regulation of the P*_gyrA126Pae_cat* and P*_gyrA121Pae_cat* carried in these plasmids was similar to that observed in the P*_topA_gfp*, P*_gyrB_gfp*, and P*_gyrB_cat* plasmid fusions (Figure 4), suggesting that the plasmid behaves as a D domain. These results were confirmed by measurement of CAT activity in both plasmids (Figure 5 E). CAT activities of the strain carrying pLGYAC126 (P*_gyrA_cat*) showed increases similar to those of their *cat*-mRNAs, as measured by qRT-PCR, both at 0.5× MIC, and 10× MIC of novobiocin. However, CAT activities of strains carrying either plasmid pLGYAC126Pae (P*_gyrA126Pae_cat*) or pLGYAC121-Pae (P*_gyrA121Pae_cat*) did not show appreciable changes after novobiocin treatment, although their *cat*-mRNAs showed a decrease. These results are compatible with the CAT protein having a higher life-time than the *cat*-mRNA. All these results support that *cis*-acting signals involved in the regulation of *gyrA* are included in the 126−nt region cloned into pLGYAC126 and that the bending is a key element for its regulation under relaxation.

## Discussion

Since the size of the *S. pneumoniae* chromosome is relatively small it probably does not require most of the nucleoid-associated proteins found in *E. coli*. Here we have shown that the organization of the chromosome in topological domains is important for its global transcription. We showed that the transcription of a heterologous P*_tc_cat* cassette depended on its location in the chromosome (Figure 3). Although topological domains were evidenced in conditions of DNA relaxation [18], we also found several instances under non-relaxation conditions (Figure 1). First, an inverse correlation was found between AT content and transcriptional activity, with D domains showing higher FU values than the genome average. Second, higher FU values were observed for the D domains when compared with those of the U domains, suggesting that D domains are transcriptionally differentiated regions and able to readily reduce their transcription under topological stress, just in opposition to U domains (Figure 1 A). Third, although low expression and high gene- lack seems globally correlated; this relationship is particularly enhanced in F domain genes. F domains are the least transcribed genes, even when compared to other non-flanking genes in the same AT range. In addition, F domains seems to be genetically unstable, therefore their genes have a higher tendency to be exchanged (Figure 1 D). Since there is a correlation between transcription and translation efficiencies [29], these results suggest that F domains play an active role as structural DNA, probably maintaining the organization of the chromosome in topological domains. In accordance, in spite of the high gene-lack index of F domains, more than half of these genes remained, suggesting that a minimal number of them are structurally necessary. The distribution of the F domains along the chromosome probably contributes to chromosome organization. Their high AT content would made them plectoneme-free regions, which form barriers between U and D domains, a role proposed for highly expressed genes in the *Caulobacter crescentus* genome [30]. Likewise, at the ends of the *Streptomyces* linear chromosome gene content is progressively lost whereas the central genome core is much more stable [31]. Whether these F domains are the binding regions for proteins stabilizing the domain structure, is a matter of future research.

Concerning the transcriptional control of the DNA topoisomerase genes (the topology-controlling enzymes), we have previously showed that the homeostatic response to DNA relaxation involves all genes coding for these *S. pneumoniae* enzymes: topoisomerase I (*topA*), topoisomerase IV (*parEC*), and DNA gyrase (*gyrA* and *gyrB*). Relaxation triggers up-regulation of gyrase and down-regulation of topoisomerase I and topoisomerase IV genes [18]. Here we have shown that the up-regulation of *gyrB* (U6 domain) and the down-regulation of *topA* (D9 domain) depended on their strategic chromosomal location (Figure 2 A). The positioning of these topology-controlling genes allows a homeostatic response, which would increase bacterial fitness under DNA topological stress. In accordance, we showed here that relaxation-mediated transcription from P*_topA_* and P*_gyrB_* promoters in a replicating plasmid differed from that of *topA* and *gyrB* in their natural chromosomal locations (Figure 4). Transcription from both promoters was down-regulated when present in the pLS1-based plasmid. This plasmid, which is commonly used for genetic studies in *S. pneumoniae*, behaves topologically as a D domain. This could be a way to neutralize the high copy number of the plasmid genes and/or favor its replication. It would be interesting to study if this down-regulated-like behavior apply to other unrelated plasmids. On the other hand, *parE-C* and *gyrA* transcription, which are located in the N6/7 and N8/9 domains, respectively seemed to be complemented with specific regulatory signals. Concerning *gyrA*, a 126−nt sequence located upstream the gene was responsible for its up-regulation under DNA relaxation. The intrinsic DNA bending present in its promoter [25] showed to be essential for P*_gyrA_* regulation, given that it showed similar up-regulation in the chromosome and in a plasmid (Figure 5). This bending act as a sensor of the supercoiling level and it behaves as an activator of transcription *per se*. This could be the consequence of a better recruitment of either the RNA polymerase complex or specific regulatory proteins. In *E. coli*, the factor for inversion stimulation (FIS) regulates the expression of DNA gyrase subunits [32], topoisomerase I [33], as well as the genes coding for other nucleoid-associated proteins involved in DNA supercoiling [34]–[36]. In addition, FIS and H-NS (histone-like nucleoid structuring protein) act controlling both the level of supercoiling and the global transcription [37], [38]. This situation seemed to be much simpler in *S. pneumoniae*, which lacks FIS and H-NS and has a less complex nucleoid-associated protein landscape. In *S. pneumoniae*, transcription of *gyrB* and *topA* is regulated by their strategic chromosomal location in topological domains, while *parEC* and *gyrA* expression is regulated by specific regulatory signals. In the case of *gyrA* a DNA bend in its promoter, which probably varies with the supercoiling status, acts as a regulator. A possibility is that P*_parE_* is regulated by a specific transcriptional regulator whose transcription depended of a bending in its promoter. This indicates that the topological organization and positioning of key genes is optimized to apply a systemic homeostatic control of chromosome supercoiling. The role of curvatures as regulators of transcription has been previously established in bacteria [39], including *S. pneumoniae* [40].

The complex homeostasis of the topological architecture of the pneumococcal chromosome relies on this interplay and, consequently, there is a hierarchical control of gene expression that is supercoiling-driven. The mechanistic bases for these observations partly rely in three different conclusions from the present study. Firs, transcriptional control is high and precise in the case of DNA topoisomerases of type II, whose action is essential for topology and cell viability. In this case, transcriptional control is mediated by a DNA bending in P*_gyrA_* and probably in the promoter of the unknown specific regulator of P*_parEC_*. Second, genes in U and D domains, which are required in case of DNA topological alterations, such as *gyrB* (U6 domain) and *topA* (D9 domain) are subjected to global topological control. And third, the remaining genes located in topological domains, which have no relevant influence on topology, are related to stress defense [18]. In conclusion, we show in this study that global and local topological signals together finely tune the chromosome architecture under a hierarchical schema to adjust transcription under physiological and stress conditions.

## Materials and Methods

### Growth and transformation of bacteria

*S. pneumoniae* was grown in a casein hydrolysate-based medium (AGCH) with 0.2% yeast extract and 0.3% sucrose as energy source and transformed with chromosomal DNA or a plasmid as described previously [41]. Transformants of *S. pneumoniae* were selected in medium containing tetracycline at 1 µg/ml for plasmids pLS1 [41] and pAST [42], or chloramphenicol at 2.5 µg/ml for chromosomal insertions (the R6 Minimal inhibitory concentration -MIC- is 1.25 µg/ml). MICs were determined in the same AGCH medium. *S. pneumoniae* R6 MIC of novobiocine was 1 µg/ml.

### DNA techniques

Chromosomal DNA and plasmids from *S. pneumoniae* were obtained as described previously [43]. Restriction endonucleases and DNA ligase were obtained from Fermentas and were used following supplier's specifications. PCR amplifications were performed using 1 U of Platinum Taq High Fidelity (Invitrogen), with an initial cycle of 30 s denaturation at 94 °C, and 30 cycles of denaturation at 94 °C for 15 s, annealing at 50 °C for 30 s and extension at 68 °C for 1 min per kb of PCR product. Primers used for PCR amplifications and sequencing are shown in Table S1. For Southern blot hybridization, 3 µg of chromosomal DNAs from the R6-CAT strains were digested with NcoI, which has a single target into *cat*. Digested DNAs were separated by 0.8% agarose gel electrophoresis, transferred to Nylon membranes and hybridized with a biothynilated 641-bp *cat* probe obtained by PCR amplification with 5′ biothynilated oligonucleotide CATRTF and CATEND2. Blots were developed with the Phototope-Star Detection Kit (New England Biolabs) following the manufacturer's instructions. To check the curvature of P*_gyrA_*, DNA fragments were run in 10% acrylamide gels in 1× TBE at 100 V either at 6°C, 20°C, or 60°C. Gels were stained with 0.5 µg/ml of ethidium bromide and images captured in a GelDoc device (BioRad Laboratories). The R^6/60^ factor was calculated as the ratio between the apparent sizes of the DNAs at 6°C and 60°C [28].

### Strains and plasmids constructions

The construction of the R6-CAT strains, which have P*_tc_cat* insertions, was carried out as indicated in Figure 2B. Firstly, three PCR products were obtained. Two, of about 1 Kb (903- to- 1433 bp), corresponding to the genes flanking the site of insertion (*sprX* and *sprY*), were obtained using primers pairs SPRXF1/−R1 and SPRYF1/−R1. Primers SPRXR1 and SPRYF1 included restriction sites for PaeI and XbaI, respectively. A third PCR fragment of 1204 bp containing P*_tc_cat* was obtained from plasmid pJS3 [22] by amplification with oligonucleotides UPTRCATXBA and CATDOWNSPH, which included XbaI and PaeI restriction sites, respectively. Secondly, the three PCR fragments were digested either with PaeI and/or XbaI and ligated. The ligation mix was then PCR-amplified with SPRXF1 and SPRYR1 to increase the amount of the ligation product. Thirdly, 0.2 µg of each ligation product were used to transform strain R6 with a chloramphenicol selection of 2.5 µg/ml. The integration of the PCR fragment by homologous recombination was checked both by amplification with primers SPRXF2 and SPRYR2, which anneal at 155 to 317 bp upstream SPRXF1 and 153 to 311 bp downstream SPRYR1, and by sequencing with the internal *cat* primers CATMED and CAT191. To fuse P*_topA_*, and P*_gyrB_* to *gfp*, fragments of 243 bp and 235 bp, amplified by PCR with primers pairs TOPAUPGFP/TOPAPROR2 and GYRBUPGFP/GYRBPROR2 were phosphorylated, digested with BamHI and ligated to plasmid pAST digested with SmaI and BamHI. Recombinant plasmids were checked by sequencing and fluorescence emission measured in a spectrofluorimeter TECAN, Inffinite F200 with emission/excitation wavelengths of 535/485 nm respectively. To fuse P*_gyrB_* and P*_parE_* to *cat*, two fragments of 244 bp and 248 bp were amplified by PCR with primers pairs GYRBUPECO/GYRBPROMR and PAREPROM1/PAREPROM2 respectively from R6 chromosome. These fragments were phosphorylated and digested with EcoRI. Moreover, *cat* cassette was amplified (658 bp) from plasmid pJS3 with primers pair CAT1/CATEND2, phosphorylated and digested with HindIII. Each fragment containing P*_gyrB_* or P*_parE_* was ligated to the fragment containing the *cat* gene and to plasmid pLS1 digested with EcoRI and HindIII. Recombinant plasmids were checked by sequencing. To construct plasmid pLGYAC126, firstly two fragments were obtained. One of 126 bp, which contained P*_gyrA_*, was amplified by PCR from the chromosome of strain R6 using primers GYRA126ECO/GYRAUPR1, and the other, of 658 bp, which contained *cat*, was amplified from plasmid pJS3 using CAT1/CATEND2. The two PCR fragments were phosphorylated, digested with EcoRI and HindIII respectively, and ligated to plasmid pLS1 digested with both enzymes. To construct plasmids pLGYAC126Pae, which has a 5 bp-insertion between the −35 and −10 boxes of P*_gyrA_*, two PCR fragments were obtained using pLGYAC126 as a substrate. One of them, obtained with primers PLS1ECO/BEND, which included EcoRI and PaeI targets respectively, and the other with PLS1HIND/BEND2, which included HindIII and PaeI targets respectively. The first fragment was digested with PaeI/EcoRI and the second with PaeI/HindIII and both were cloned into pLS1 digested with EcoRI and HindIII. To construct plasmid pLGYAC121Pae, two fragments were amplified by PCR: one from pLGYAC126Pae with primers PLS1ECO/BEND1 which contained EcoRI and PaeI targets respectively, and the other from pLGYAC126 with primers BEND4/PLS1HIN, which contained PaeI and HindIII targets, respectively. Fragments were cut with their respective restriction enzymes and cloned in pLS1 digested with EcoRI and HindIII. Plasmid constructions were checked by digestion with PaeI and sequencing with oligonucleotide CATMED.Sequences of all oligonucleotides used in this work are available in Table S1.

### RNA techniques

Synthesis of cDNAs from 5 µg of total RNA was performed as previously described [24]. cDNAs obtained were subjected to quantitative qRT-PCR (Chromo 4, BioRad) in 20 µl reactions containing 2 µl of cDNA, 0.4 µM of each specific primer, and 10 µl of LightCycler FastStart Universal A SYBR Green Master (Roche). Amplification was achieved with 42 cycles of a three-segment program: denaturation (30 s at 94 °C), annealing (30 s at 45–56 °C), and elongation (30 s at 68°C). Oligonucleotide pairs used were: GYRARTF/GYRARTR (*gyrA*); GYRBRTF/GYRBRTR (*gyrB*); TOPARTF/TOPARTR (*topA*); PARE214/PARE274R (*parE*) [24]; CATRTF/CATRTR (*cat*); GFPRTF/GFPRTR (*gfp*). To normalize the three independent cDNA replicate samples, values were divided by those obtained of the amplification of an internal fragment of 16SrDNA [18].

### CAT activity measurement

Preparation of crude extracts and CAT activity determinations were carried out as described previously [22].

### Microarray data normalization and analysis

High density *Streptococus pneumoniae* expression arrays from Agilent were processed at the Functional Genomics Core Facility, Institut de Recerca Biomèdica, Barcelona. Arrays were designed including 1 or 2 copies of 17 oligonucleotide probes (average size of 60 nucleotides) for each 2,037 protein coding genes from *S. pneumoniae* R6. cDNA library preparation and amplification was performed from 10 ng of R6 genomic DNA using WGA2 (Sigma Aldrich) with 10 cycles amplification. Labelling of 250 ng of cDNAs with Cy3 was performed by ULS. Hybridization was made following the Agilent Oligo Array-based CGH for genomic DNA analysis ULS protocol. Microarrays were scanned on a Roche MS200 scanner at 2 µm resolution and raw data were extracted using Feature Extraction software v11.5. Raw data were RMA normalized using Partek Genomics suite 6.5 (6.11.0207). The median of each group of 17 probes in each block of the array were considered. Each microarray experiment was carried out in duplicate.

### Microrray data accession number

All microarray data has been uploaded to the GEO database (http://www.ncbi.nlm.gov/geo), with code GSE58186.

### Genomic analysis

The 12 *S. pneumoniae* genome sequences analyzed (11 clinical isolates plus strain R6) were taken from the NCBI database and compared with BLAST. A gene was considered to have an equivalent in other strain when their polypeptide products shared ≥80% identity over ≥80% of the sequence length. Average fluorescence values at time 0 min used were taken from three independent replicates of R6 strain [18] and are available at the Array Express (EBI, UK) database via accession number E-MTAB-141. CAI values were downloaded from the HGT-DB resource [44].

## Supporting Information

Figure S1 **DNA sequence of the P** ***_tc_cat*** **cassette showing their main transcriptional and translational features.** Oligonucleotides used to amplify the cassette are indicated with double- underlining. The −35 and −10 boxes of the P_t_ and P_c_ promoters, the nucleotide were transcription is initiated (+1), and the ATG initiation codon are showed in boldface and underlined. The two transcription terminators (Tr) are underlined. The NcoI target is shown in boldface.
(DOCX)

Figure S2 **Transcriptional response to relaxation by novobiocin measured by qRT-PCR.** Exponentially growing cultures of the R6-CAT strains in AGCH medium supplemented with 0.3% sucrose and 0.2% Yeast Extract at OD_620 nm_ = 0.4 were treated with novobiocin at 10× MIC. Total RNA was isolated; cDNA was synthesized and subjected to qRT-PCR. To normalize the three independent replicate samples, values were divided by those obtained from an internal fragment of the16S rRNA, whose absolute qRT-PCR values are shown. Transcription represented the mean of qRT-PCR values of three independent replicates ± SD.
(TIF)

Figure S3 **DNA sequence of the 5′** ***gyrB*** **region and localization of the transcription initiation site.** Sequenase reactions using plasmid pGYRN5 as the template and gyrB22 as the primer provided a reference sequence ladder. G, A, T, and C indicated the dideoxynucleotides used during the sequencing assay. For primer extension experiments, RNAs obtained from *E. coli* XL1-Blue containing either pGYRN5 (lane 1, 15 µg of RNA) or pEMBL18^+^ (lane 2, 15 µg of RNA) were used. The arrow indicates the direction of electrophoresis. The −35 region, the first nucleotide of the mRNA (+1), and putative ribosome-binding site (RBS) are framed. The double-strand DNA sequence of the 5′ *gyrB* region and the deduced amino acid sequence are shown. The construction of pGYRN5 has been described elsewhere [19].
(TIF)

Figure S4 **Deletions from the 5′-end of the P** ***_gyrA_cat*** **fusion eliminate progressively the curvature present in the promoter region of** ***gyrA*** **.** (A) DNA sequence of the P*_gyrA_cat* cassette showing their main transcriptional and translational features. The −35 and extended −10 boxes of the P*_gyrA_* promoter, the nucleotides the 5′ deletion ends (double underlined), and the nucleotide were transcription is initiated (+1) are shown. (B) Mobility of fragments at three temperatures. Fragments from the diverse plasmids were obtained after PCR amplification with oligonucleotide PUC19R, located at 85 bp of the 5′ end of the sequence shown in A) and CAT9 (located at 24 nt of the first *cat* nucleotide). (C) Determination of the apparent length of each fragment. Sizes were of 430, 343, 310, 287, 230 and 220 for plasmids carrying P*gyrA* regions −269, −182, −149, −126, −69, and −59, respectively. Electrophoresis was carried out in 3.5% polyacrylamide gels and bands were observed after ethidium bromide staining. A 100 bp DNA ladder was used as a molecular weight marker (Mw).
(TIF)

Table S1 **Oligonucleotides used in this study.**
(DOCX)
