# Supplementary material for: Role of Global and Local Topology in the Regulation of Gene Expression in Streptococcus pneumoniae
Source: PLoS One. 2014 Jul 14;9(7):e101574. doi: 10.1371/journal.pone.0101574 (PMC4096756; doi:10.1371/journal.pone.0101574)
Supplement: Figure S1 — DNA sequence of the P tccat cassette showing their main transcriptional and translational features. Oligonucleotides used to amplify the cassette are indicated with double- underlining. The −35 and −10 boxes of the Pt and Pc promoters, the nucleotide were transcription is initiated (+1), and the ATG initiation codon are showed in boldface and underlined. The two transcription terminators (Tr) are underlined. The NcoI target is shown in boldface. (DOCX) [file pone.0101574.s001.docx]

⇒ UPTRCAT

GACGGAGCTTATCAACGTAGTAAGCGTGGAACAAAGAACGAAGAGAGATAAAAAGTTGAT

**Tr**

CTTTGTGAAAACTACAGAAAGTAAAGAATGAAAAGA**GTAATGCTAA**CA**TAGCATTAC**GGA

**-35P_t_** **-10P_t_**

TTTTATGACCGATGATGAAGAAAAGAAT**TTGAAA**CTTAGTTTATATGTGG**TAAAAT**GTTT

TAATCAAGATAAGAAAGAAAAGGATTTTTCGCTACGCTCAAATCCTTTAAAAAAACACAA

AAGACCACATTTTTTAATGTGGTCTTTTATTCTTCAACTAAAGCACCCATTAGTTCAACA

**-35P_c_**

AACGAAAATTGGATAAAGTGGGATATTTTTAAAATATATATTTATGTTACAGTAATA**TTG**

**-10P_c_ +1**

**AC**TTTTAAAAAAGGATTGAT**TCTAAT**GAAGAA**A**GCAGACAAGTAAGCCTCCTAAATTCAC

TTTAGATAAAAATTTAGGAGGCATATCAA**ATG**AACTTTAATAAAATTGATTTAGACAATT

*cat* M N F N K I D L D N

GGAAGAGAAAAGAGATATTTAATCATTATTTGAACCAACAAACGACTTTTAGTATAACCA

W K R K E I F N H Y L N Q Q T T F S I T

CAGAAATTGATATTAGTGTTTTATACCGAAACATAAAACAAGAAGGATATAAATTTTACC

T E I D I S V L Y R N I K Q E G Y K F Y

CTGCATTTATTTTCTTAGTGACAAGGGTGATAAACTCAAATACAGCTTTTAGAACTGGTT

P A F I F L V T R V I N S N T A F R T G

ACAATAGCGACGGAGAGTTAGGTTATTGGGATAAGTTAGAGCCACTTTATACAATTTTTG

Y N S D G E L G Y W D K L E P L Y T I F

ATGGTGTATCTAAAACATTCTCTGGTATTTGGACTCCTGTAAAGAATGACTTCAAAGAGT

D G V S K F S G I W T P V K N D F K E T

TTTATGATTTATACCTTTCTGATGTAGAGAAATATAATGGTTCGGGGAAATTGTTTCCCA

F Y D L Y L S D V E K Y N G S G K L F P

AAACACCTATACCTGAAAATGCTTTTTCTCTTTCTATTATT**CCATGG**ACTTCATTTACTG

K T P I P E N A F S L S I I P W T S F T

GGTTTAACTTAAATATCAATAATAATAGTAATTACCTTCTACCCATTATTACAGCAGGAA

G F N L N I N N N S N Y L L P I I T A G

AATTCATTAATAAAGGTAATTCAATATATTTACCGCTATCTTTACAGGTACATCATTCTG

K F I N K G N S I Y L P L S L Q V H H S

TTTGTGATGGTTATCATGCAGGATTGTTTATGAACTCTATTCAGGAATTGTCAGATAGGC

V C D G Y H A G L F M N S I Q E L S D R

CTAATGACTGGCTTTTATAATATGAGATAATG**CCGACTGTA**CTTTT**TACAGTCGG**TTTTC

P N D W L L **Tr**

TAATGTCACTAACCTGCCCCGTTAGTTGAAGAAGGTTTTTATATTACAGCTCCAGATCCA

TATC

⇐CATDOWN
