# Supplementary figures and images for: Role of Global and Local Topology in the Regulation of Gene Expression in Streptococcus pneumoniae
Source: PLoS One. 2014 Jul 14;9(7):e101574. doi: 10.1371/journal.pone.0101574 (PMC4096756; doi:10.1371/journal.pone.0101574)

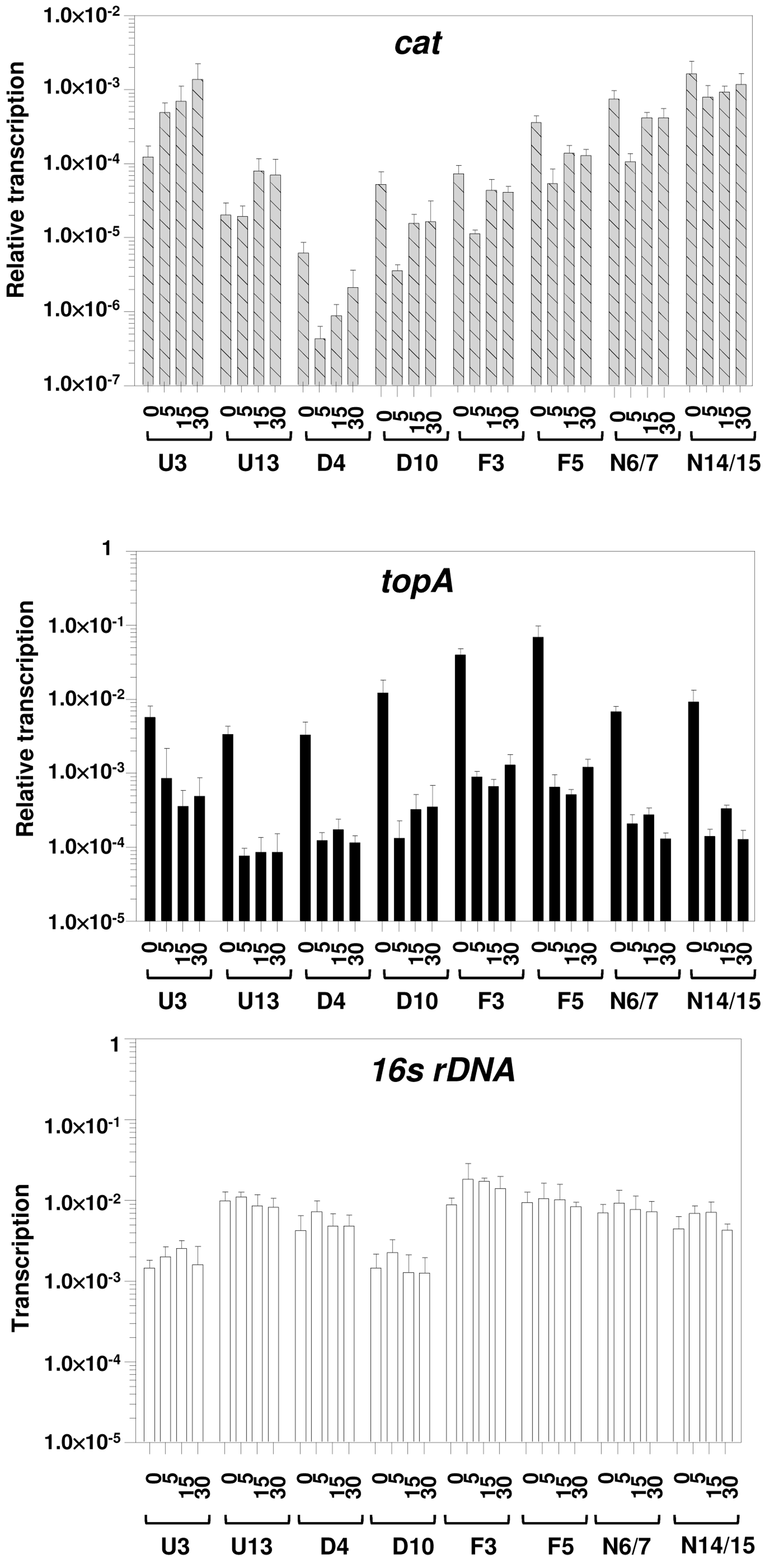

Supplement: Figure S2 — Transcriptional response to relaxation by novobiocin measured by qRT-PCR. Exponentially growing cultures of the R6-CAT strains in AGCH medium supplemented with 0.3% sucrose and 0.2% Yeast Extract at OD620 nm = 0.4 were treated with novobiocin at 10× MIC. Total RNA was isolated; cDNA was synthesized and subjected to qRT-PCR. To normalize the three independent replicate samples, values were divided by those obtained from an internal fragment of the16S rRNA, whose absolute qRT-PCR values are shown. Transcription represented the mean of qRT-PCR values of three independent replicates ± SD. (TIF) [file pone.0101574.s002.tif]

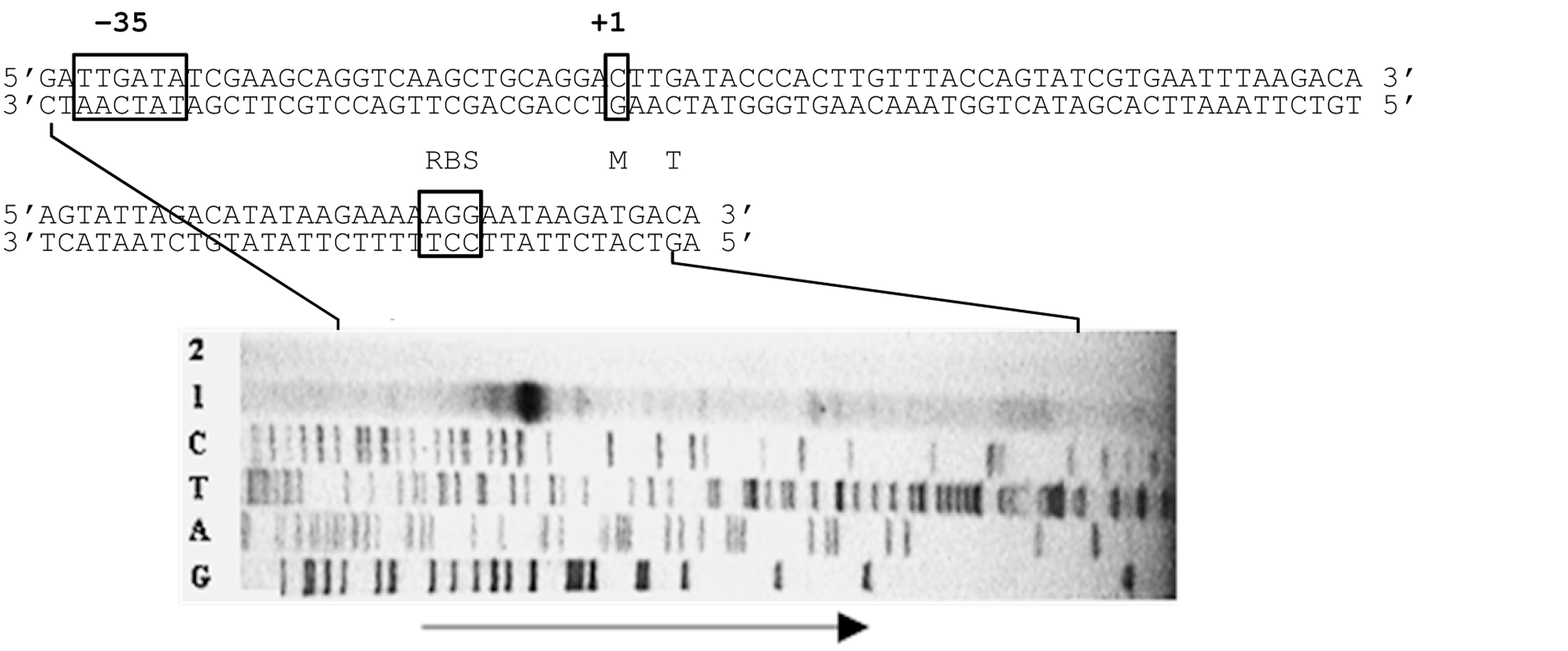

Supplement: Figure S3 — DNA sequence of the 5′ gyrB region and localization of the transcription initiation site. Sequenase reactions using plasmid pGYRN5 as the template and gyrB22 as the primer provided a reference sequence ladder. G, A, T, and C indicated the dideoxynucleotides used during the sequencing assay. For primer extension experiments, RNAs obtained from E. coli XL1-Blue containing either pGYRN5 (lane 1, 15 µg of RNA) or pEMBL18+ (lane 2, 15 µg of RNA) were used. The arrow indicates the direction of electrophoresis. The −35 region, the first nucleotide of the mRNA (+1), and putative ribosome-binding site (RBS) are framed. The double-strand DNA sequence of the 5′ gyrB region and the deduced amino acid sequence are shown. The construction of pGYRN5 has been described elsewhere [19]. (TIF) [file pone.0101574.s003.tif]

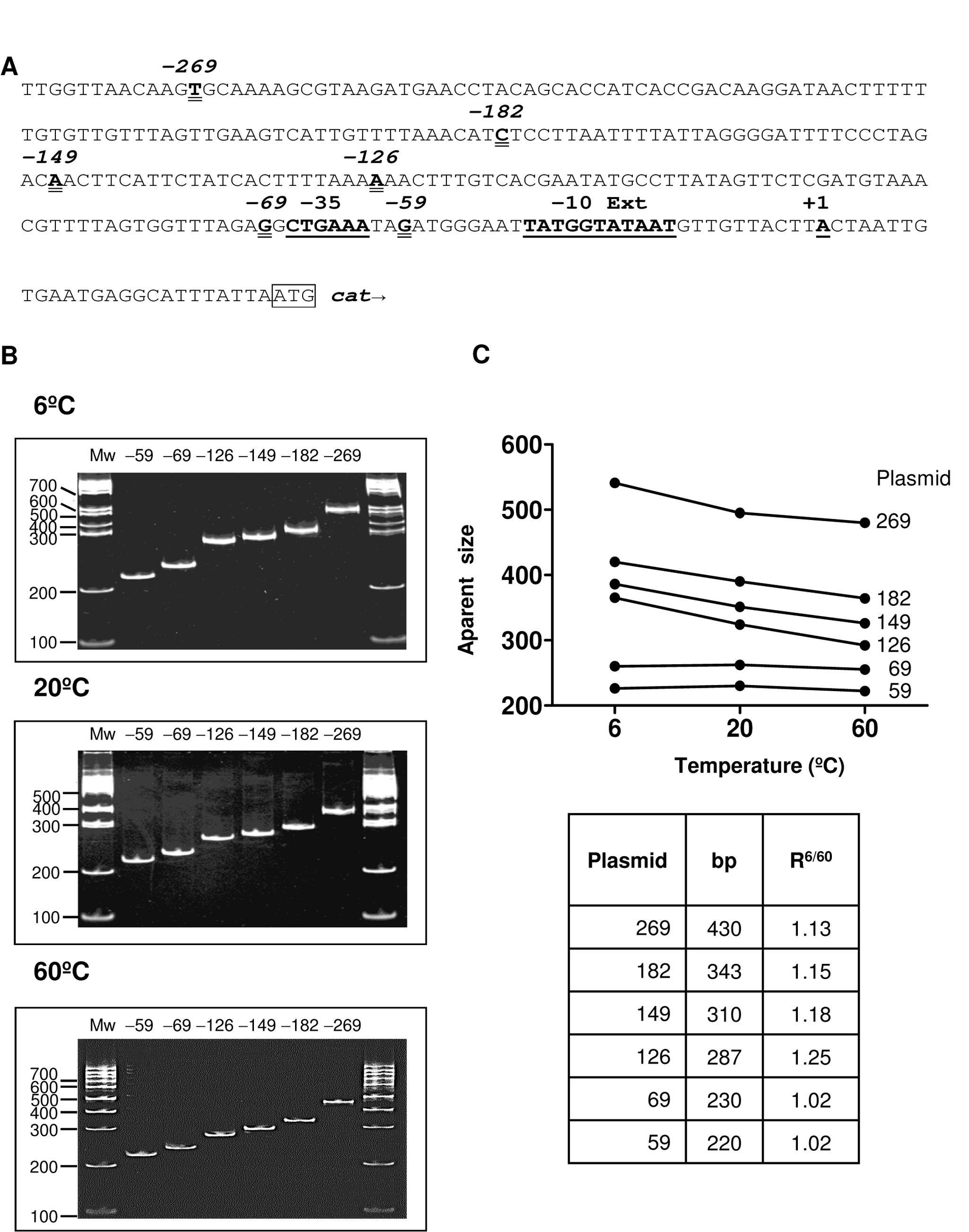

Supplement: Figure S4 — Deletions from the 5′-end of the P gyrAcat fusion eliminate progressively the curvature present in the promoter region of gyrA . (A) DNA sequence of the PgyrAcat cassette showing their main transcriptional and translational features. The −35 and extended −10 boxes of the PgyrA promoter, the nucleotides the 5′ deletion ends (double underlined), and the nucleotide were transcription is initiated (+1) are shown. (B) Mobility of fragments at three temperatures. Fragments from the diverse plasmids were obtained after PCR amplification with oligonucleotide PUC19R, located at 85 bp of the 5′ end of the sequence shown in A) and CAT9 (located at 24 nt of the first cat nucleotide). (C) Determination of the apparent length of each fragment. Sizes were of 430, 343, 310, 287, 230 and 220 for plasmids carrying PgyrA regions −269, −182, −149, −126, −69, and −59, respectively. Electrophoresis was carried out in 3.5% polyacrylamide gels and bands were observed after ethidium bromide staining. A 100 bp DNA ladder was used as a molecular weight marker (Mw). (TIF) [file pone.0101574.s004.tif]
