# Supplementary material for: Role of Global and Local Topology in the Regulation of Gene Expression in Streptococcus pneumoniae
Source: PLoS One. 2014 Jul 14;9(7):e101574. doi: 10.1371/journal.pone.0101574 (PMC4096756; doi:10.1371/journal.pone.0101574)
Supplement: Table S1 — Oligonucleotides used in this study. (DOCX) [file pone.0101574.s005.docx]

| **Primer name** | **Sequence (5’→ 3’)^a, b^** | **Nucleotide positions^c^** |
| --- | --- | --- |
| **Amplification and sequence of *cat* gene** | | |
| UPTRCATXBA | gcgcgtctaGACGGAGCTTATCAACGTAGT | -449 to -429 of *cat* |
| CATDOWNSPH | gcgcggcatgcGATATGGATCTGGAGCTGTAA | Complementary to 734 to 754 of *cat* |
| CATMED | CCTAACTCTCCGTCGCTATTG | Complementary to 212 to 232 of *cat* |
| CAT191 | GTGATGGTTATCATGCAGG | 574 to 592 of *cat* |
| CAT1 | ATGAACTTTAATAAAATTGATTTAGAC | 1 to 26 of *cat* |
| CATEND2 | gcgcgaagctTCTCATATTATAAAAGCCAGTC | Complementary to 636 to 657 of *cat* |
| **Fusions of P*_gyrB_* and P*_topA_* to *gfp* gene** | | |
| TOPAUPGFP | cgcgcgggaTCCAGTATATCAAGGATAAGG | -243 to -222 of *topA* |
| TOPAPROR2 | AAGTAATAAACTTTATAAAGTGTAAACC | Complementary to -45 to -18 of *topA* |
| GYRBUPGFP | cgcgcggatCCTCTATAGCAGCTTATTTTAC | -235 to -213 of *gyrB* |
| GYRBPROR2 | TATATGTCTAATACTTGTCTTAAATTC | Complementary to -42 to -15 of *gyrB* |
| **Fusions of P*_gyrB_* and P*_parE_* to *cat* gene** | | |
| PAREPROM1 | gcgcggaattCATGCTCGCGTAGATTGATT | 237 to 218 |
| PAREPROM2 | AAGGAACCTCCTATTCGTTCATC | Complementary to -23 to -1 of *parE* |
| GYRBUPECO | cgcgcgaatTCTCTATAGCAGCTTATTTTAC | -233 to -213 of *gyrB* |
| GYRBPROMR | CTTATTCCTTTTTCTTATATG | Complementary to -21 to -1of *gyrB* |
| **Determination of the transcription initiation site of *gyrB*** | | |
| GYRB22 | CGCATACGAACAGCCTCTAAGCCCT | Complementary to 64 to 89 of gyrB |
| **Fusions of P*_gyrA_* to *cat* gene** | | |
| GYRA126ECO | gcgcgcgaattcAAACTTTGTCACGAATATGCC | -126 to -104 of *gyrA* |
| GYRAUPR1 | TAATAAATGCCTCATTTCACAA | Complementary to -22 to -1 of *gyrA* |
| BEND1 | gcgcgcatgCATCTATTTCAGCCTCTAAAC | Complementary to -84 to -54 of *gyrA* |
| BEND2 | gcgcgcatgcGGAATTTATGGTATAATGTTG | -54 to -33 of *gyrA* |
| PLS1ECO | GGGAGAGAGTTCAAAATTGA | -37 to -17 EcoRI |
| PLS1HIN | GGTTAGTTGTTTCACGTATCGG | Complementary to 44 to 66 of HindIII |
| **qRT-PCR** | | |
| CATRTF | ATACCGAAACATAAAACAAGAAGG | 113 to 137 of *cat* |
| CATRTR | TACAGGAGTCCAAATACCAGAGAA | Complementary to 288 to 312 of *cat* |
| GFPRTF | TTGCACTACTGGAAAACTACCTG | 140 to 163 of *gfp* |
| GFPRTR | TCGGGCATGGCACTCTTG | 251 to 269 of *gfp* |
| **Construction of U3 strain** | | |
| SPR0152F1 | GACACCAATCCCCATGCAGAG | -203 to -182 of *spr0152* |
| SPR0152F2 | ACCAGCACCGTGTTTAGTTCC | -380 to -359 of *spr0152* |
| SPR0152R1 | AAGCTTTCCGCTCTAATTGCC | Complementary 278 to 299 *spr0152* |
| SPR0153F1 | gcgcgtctaGATTTTGTAACTGGGGTGGCC | -597 to -576 of *spr0153* |
| SPR0153R1 | ATCCCGATAGACTGCCTTTGA | Complementary 560 to 581 *spr0153* |
| SPR0153R2 | GCTGTTGCGACGACCTCCATA | Complementary 784 to 805 *spr0153* |
| **Construction of U13 strain** | | |
| SPR1525F1 | GACAGGGGCATTCAAATCACA | 101 to 122 of *spr1525* |
| SPR1525F2 | CTCATTCCAGTGTTTCGCCTTG | -223 to -201 of *spr1525* |
| SPR1525R1 | gcgcggcatgCACTTATAACCTCTATATTCTC | Complementary to 987 to 1009 of *spr1525* |
| SPR1524F1 | gcgcgtctaGAGGGTATGCAGTTACTTTA | -90 to -69 of *spr1524* |
| SPR1524R1 | TACATCAGCTCCGAGCACAGC | Complementary to 789 to 810 of *spr1524* |
| SPR1524R2 | AAATCCATCTAGCCCTCCACG | Complementary to 960 to 981 of *spr1524* |
| **Construction of D4 strain** | | |
| SPR0270F1 | CGAACGAGCCTTTATCGCCCT | 849 to 870 of *spr0270* |
| SPR0270F2 | AGGCAGCTACAGACTTGGATT | 1087 to 1108 of *spr0270* |
| SPR0270R1 | gcgcggcatgCCTTCTTTTTCCTTGTAGTTA | Complementary to -48 to -27 of *spr0270* |
| SPR0271F1 | gcgcgtctagaCCCAAAAATCTACCGAAGTT | -245 to -225 of *spr0271* |
| SPR0271R1 | GGAAGTCTGGGTCTACTTGAAG | Complementary to 724 to 746 of *spr0271* |
| SPR0271R2 | CAGCCACATTTGTAAGCGACT | Complementary to 956 to 977 of *spr0271* |
| **Construction of D10 strain** | | |
| SPR1323F1 | TCCACAAGCGTTGTAGGCACC | 960 to 981 of *spr1323* |
| SPR1323F2 | GGCAAGAAGAAGAGGTCTGTC | 1298 to 1319 of *spr1323* |
| SPR1323R1 | gcgcggcatgCTTGAAAACAGGATAAATGGG | Complementary to -128 to -107 of *spr1323* |
| SPR1324F1 | gcgcgtctagaGTAAGGGCTTCAATTTAGATG | -70 to -49 of *spr1342* |
| SPR1324R1 | GATGCCCTCCAAACTTTCGAC | Complementary to 837 to 858 of *spr1324* |
| SPR1324R2 | AACGCCATCTGCCTCTTCGAT | Complementary to 1169 to 1190 of *spr1324* |
| **Construction of F3 strain** | | |
| SPR0938F1 | GAAGCCCAATATTAGCTGGAT | -166 to -145 of *spr0938* |
| SPR0938F2 | GAGATGGCTCAGTGGCTCTTT | -453 to -432 of *spr0938* |
| SPR0938R1 | gcgcggcatGCTGTTATTCTCTATATCC | Complementary to 951 to 970 of *spr0938* |
| SPR0939F1 | GTACAACGACTAGTTCAAAAGC | -352 to -330 of *spr0939* |
| SPR0939R1 | TCTCACCTCCGCCTCTATTCC | Complementary to 1059 to 1078 of *spr0939* |
| SPR0939R2 | CTGGCGTTGGATCGGGTAGA | Complementary to 1250 to 1270 of *spr0939* |
| **Construction of F5 strain** | | |
| SPR1281F1 | gcgcgtctagaGTAAGTAAGCATTATGGTCAT | -108 to -87 of *spr1281* |
| SPR1281R1 | ACAAACTTACCGTATTGTGGG | Complementary to 970 to 991 of *spr1281* |
| SPR1281R2 | AAATACCAAATACCAAGCCAC | Complementary to 1225 to 1246 of *spr1281* |
| SPR1282F1 | ATGCGTGATGCCAACCGTGGT | 1 to 21 of *spr1282* |
| SPR1282F2 | AGTAGGAGAAAACTCAATTCC | -244 to -223 of *spr1282* |
| SPR1282R1 | gcgcggcatgcTCATGGTTTGCTCTTTGATTC | Complementary to 1065 to 1086 of *spr1282* |
| **Construction of NR6/7 strain** | | |
| SPR0849F1 | GACATTCAACTGAGTCATCCAG | 10 to 40 of *spr0849* |
| SPR0849F2 | GGAGGAGCATGCCAGTTTCTC | -158 to -137 of *spr0849* |
| SPR0849R1 | gcgcggcaTGCCTAATCGTTGTCTCAGACC | Complementary to 1049 to 1071 of *spr0849* |
| SPR0850F1 | gcgcgtctAGAACTTTTATGTGGCTGATGAC | -82 to -59 of *spr0850* |
| SPR0850R1 | GCTGAGCTCTTTAGTCCCAGC | Complementary to 897 to 918 of *spr0850* |
| SPR0850R1 | GAAGGCTTGCGTCAAGGTGTG | Complementary to 1214 to 1235 of *spr0850* |
| **Construction of NR14/15 strain** | | |
| SPR1794F1 | CAGCTGACCTGAGGCAAACTC | 121 to 142 of *spr1794* |
| SPR1794F2 | AGCCCTTCTTAACTCCCTATC | -215 to -194 of *spr1794* |
| SPR1794R1 | gcgcggcatgCAATAGAACTGACAAACCCTGT | Complementary to 1160 to 1181 of *spr1794* |
| SPR1793F1 | gcgcgtctagACAGTGTTTTGAACCACCAGC | -79 to -58 of *spr1793* |
| SPR1793R1 | GGGAGTCGGGAACTCGGCAAC | Complementary to 936 to 957 of *spr1793* |
| SPR1793R2 | GTCGCACCCAATTCACGTGCA | Complementary to 1214 to 1235 of *spr1793* |

^a^ In lower case are indicated bases added to the annealing sequence. ^b^ The underlined sequences correspond to restriction targets. ^c^ Nucleotide numbering refers to the genes of the *S. pneumoniae* R6 sequence except for primers PLS1ECO and PLS1HIN for which the numbering refers to the sequence of plasmid pLS1. The first nucleotide of the gene is considered as the nt 1.
